# Application of Hydrophilic Silanol-Based Chemical Grout for Strengthening Damaged Reinforced Concrete Flexural Members

**DOI:** 10.3390/ma7064823

**Published:** 2014-06-23

**Authors:** Hyunjin Ju, Deuck Hang Lee, Hae-Chang Cho, Kang Su Kim, Seyoon Yoon, Soo-Yeon Seo

**Affiliations:** 1 Department of Architectural Engineering, University of Seoul, 163 Seoulsiripdae-ro, Dongdaemun-gu, Seoul 130-743, Korea; E-Mails: fis00z@uos.ac.kr (H.J.); dklee@uos.ac.kr (D.H.L.); haechang41@naver.com(H.-C.C.); 2 School of Engineering, University of Aberdeen, 361 Fraser Noble Building, King’s College, Aberdeen AB24 3UE, UK; E-Mail: yoonseyoon@gmail.com; 3 Department of Architectural Engineering, Korea National University of Transportation, 50 Daehak-ro Chungju-si, Chngbuk 380-702, Korea; E-Mail: syseo@ut.ac.kr

**Keywords:** strengthening, HCGS, partial interaction, flexural behavior, silanol

## Abstract

In this study, hydrophilic chemical grout using silanol (HCGS) was adopted to overcome the performance limitations of epoxy materials used for strengthening existing buildings and civil engineering structures. The enhanced material performances of HCGS were introduced, and applied to the section enlargement method, which is one of the typical structural strengthening methods used in practice. To evaluate the excellent structural strengthening performance of the HCGS, structural tests were conducted on reinforced concrete beams, and analyses on the flexural behaviors of test specimens were performed by modified partial interaction theory (PIT). In particular, to improve the constructability of the section enlargement method, an advanced strengthening method was proposed, in which the precast panel was directly attached to the bottom of the damaged structural member by HCGS, and the degree of connection of the test specimens, strengthened by the section enlargement method, were quantitatively evaluated by PIT-based analysis.

## 1. Introduction

Suitable maintenance is typically required for reinforced concrete structures, when they lose their structural or durability performance during their intended service life due to unexpected excessive loading, durability damage of the concrete or corrosion of the steel reinforcement [1]. In addition, appropriate reinforcement is also often required when remodeling or expanding existing buildings. The typical methods commonly used for the structural strengthening of existing concrete structures include the section enlargement, steel plate or FRP sheet bonding method, and external prestressing methods. Recently cement based composites (FRCM materials) have been proposed, as described in ACI 549 and in technical papers [2,3,4,5,6,7]. While there are some differences depending on the method applied, it is well known that one of the most important key factors for successful strengthening of concrete structures is the bond performance of the binding material, which greatly affects the structural behavior of the strengthened member [8,9,10,11,12,13,14,15,16,17].

Epoxy-based materials are widely used as the binding material for structural strengthening. The material properties of the epoxy resin, however, are easily degraded on contact with water and it even loses its original function. It has also been consistently pointed out that water-soluble epoxy is not often suitable for structural strengthening because its binding capacity is insufficient even after hardening [18]. In addition, a primer generally needs to be applied to the target member before applying epoxy to it, to ensure adequate bonding of the epoxy resin; this reduces the constructability, and in many cases, the hardening of the epoxy reduces the strength and hardness, which results in delamination at the interface with the base member [19]. To overcome the limitations of the existing epoxy materials described above, an alternative material with improved bonding performance, as well as a method that is capable of improving the constructability, needs to be developed.

In this study, the bonding mechanism of hydrophilic chemical grout using silanol (HCGS), which is capable of strong chemical bonding through ionic bonding between the silanol and concrete was investigated, and material tests were conducted to evaluate the performances of HCGS as an alternative binding material. Also, the structural performances of the members strengthened by the section enlargement method utilizing HCGS were evaluated based on experiment and comprehensive analysis. In the experimental program, one control specimen was an undamaged reinforced concrete (RC) beam specimen, and three damaged RC beam specimens were strengthened by the section enlargement method. For strengthening of the damaged specimens, HCGS were applied to two of the specimens, while a conventional type of chemical anchor (*i.e.*, shear connectors) was applied to the third.

## 2. Development and Properties of HCGS

### 2.1. Development of HCGS

**Figure 1:**
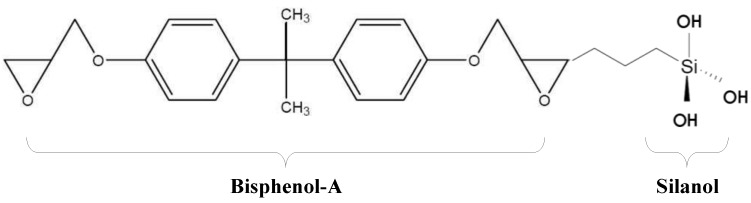
Chemical structure of hydrophilic chemical grout using silanol (HCGS).

**Figure 2:**
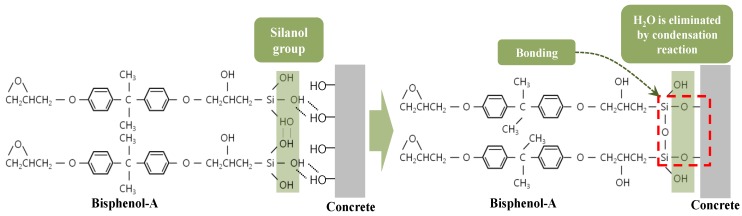
Chemical bonding mechanism between HCGS and concrete.

As shown in Figure 1, HCGS can be chemically synthesized by combining the silanol group (Si-OH) with a bisphenol-A-type epoxy resin. HCGS with such a chemical structure shown in Figure 1 is known to have superior mechanical performances, electrical properties, chemical resistance, weathering and corrosion resistance [20,21,22]. As HCGS can provide strong chemical welding through ionic bonding with the silanol group, it forms a strong bonding mechanism when used as a binding material for structural strengthening of damaged members. Figure 2 shows the reaction mechanism of HCGS to concrete. The silanol group (Si-OH) in the HCGS and the inorganic ions in concrete are combined in a condensation reaction, in which the water molecules (H_2_O) are separated, and the covalent ionic bonding of Si-O-concrete strongly binds the HCGS with concrete. In addition, since the hydrophilic characteristic of HCGS allows mutual transference of water ions, which can prevent any undesirable stress at the interface and the resultant detachment, it allows sustained stability and high durability. On this basis, it can be stated that HCGS is capable of overcoming the limitations of conventional epoxy materials.

### 2.2. Material Characteristics of HCGS

**Figure 3:**
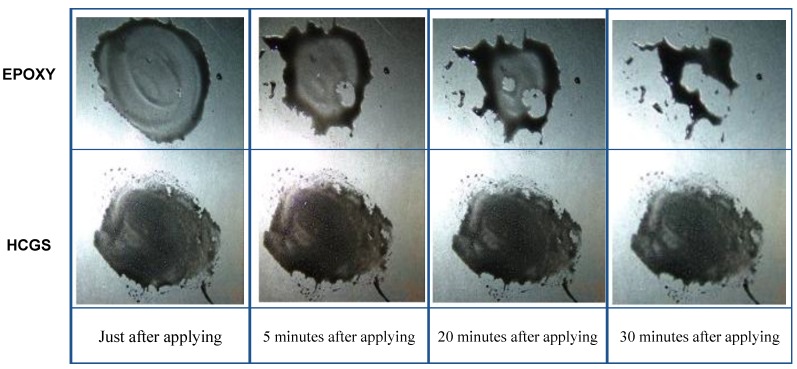
Comparison of surface tension force developed in HCGS and epoxy.

Material tests were performed, based on KS F 4042 [23] or other standards shown in Table 1, to identify the material characteristics and performances of the HCGS introduced in this study, and the key material test results are shown in Table 1 compared with those of a conventional epoxy product that has been mass-produced by “S” Company (Seoul, Korea). Table 1 shows that the basic mechanical properties of the HCGS (e.g., compressive strength, bond strength, and modulus of rupture) are superior to those of the conventional epoxy product. The HCGS also shows better performances compared to the conventional epoxy product in terms of durability, such as alkali resistance and carbonation resistance. In particular, since the bond strength of the HCGS is superior to that of the conventional epoxy, it can be considered that HCGS can replace conventional epoxy as a binding material for structural strengthening. In addition, as shown in Figure 3, the surface tension of the HSCG is significantly lower than that of conventional epoxy, which means that the bonding performance of HCGS is superior to typical epoxy. Also, because of this, primer work is not required for the application of HCGS. On this basis, superior bonding performance can be achieved without primer application before resin application, thereby ensuring far superior constructability compared with other epoxy products.

**Table 1:** Comparison of material properties between hydrophilic chemical grout using silanol (HCGS) and epoxy.

Tests		HCGS	Epoxy	Ref. Standards
Compressive strength (MPa)		59.1	51.3	ASTM C109 [24]
Modulus of rupture (MPa)		20.1	8.2	ASTM C293 [25]
Bond strength	Standard (MPa)	3.6	1.9	ASTM C1583 [26]
	Cyclic heat temperature (MPa)	2.1	1.5	
Alkali resistance (Compressive Strength) (MPa)		57.4	50.4	ASTM C227 [27]
Water permeability (g)		1	2	ISO.12572 [28]
Water absorption coefficient (kg/m^2^∙h^0.5^)		0.06	0.11	ISO.15148 [29]
Water vapor transmission, S_d_ (m)		1.2	1.7	ASTM E 96 [30]
Chloride ion permeation resistance (Coulombs)		427	3926	ASTM C1202 [31]
Changing rate of length (%)		−0.06	−0.10	ASTM C157 [32]
Carbonation depth (mm)		1.9	3.2	ISO DIS 1920-12.2 [33]

## 3. Experimental Program

**Figure 4:**
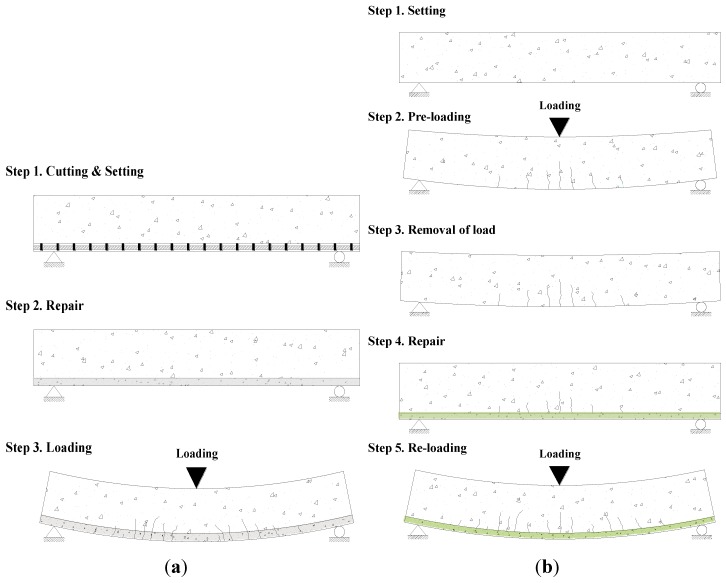
Schematic description of pre-damaging and post-strengthening loading test: (**a**) existing testing method; (**b**) proposed testing method.

As shown in Figure 4a, experiments in previous studies were carried out by strengthening undamaged concrete members. With such experimental methods, however, it is difficult to quantitatively evaluate strengthening efficiency. As shown in Figure 4b, in this study, therefore, the reinforced concrete beam members were damaged in advance through preloading before performing the flexural strengthening, which is considered to be similar to real situations.

### 3.1. Specimens and Material Characteristics

**Figure 5:**
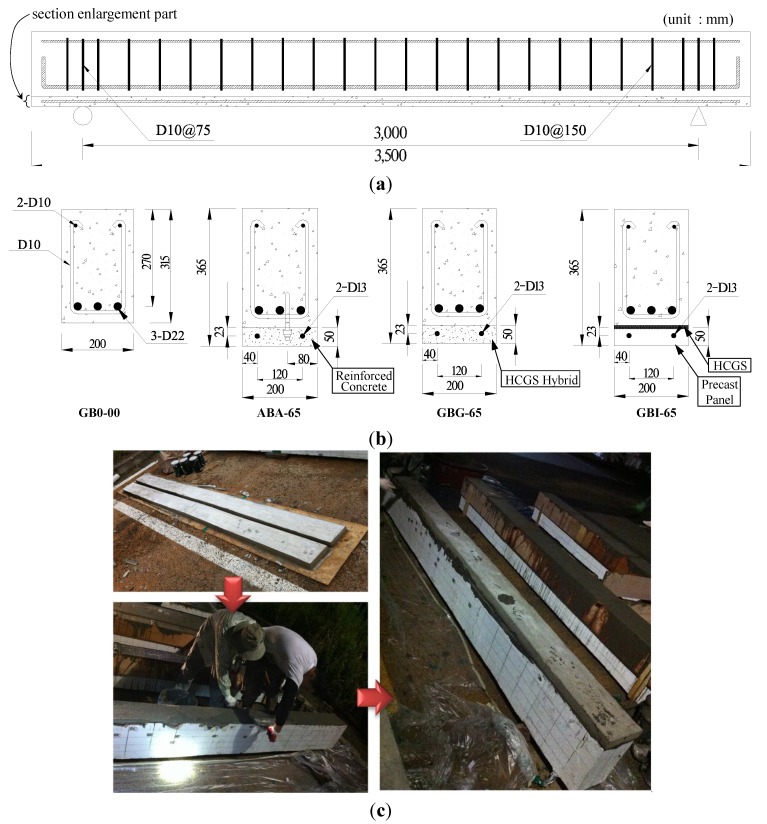
Details of test specimens: (**a**) geometry and reinforcing details of specimens; (**b**) sectional details of specimens; (**c**) precast panel strengthening process.

In this study, the tests were conducted on one reinforced concrete beam without strengthening, the control specimen, and three specimens strengthened by the section enlargement method applying HCGS after preloading. To simulate the actual strengthening situation, the strengthened specimens were preloaded up to 65% of the flexural capacity of the control specimen before strengthening them. The preload was planned at about 60% of the flexural capacity of the control specimen, which was considered to be about the service load level of the member. After testing the control specimen GB0-00, however, the preload was increased up to 65% of the flexural capacity of the control specimen, because under the 60% preload the damage was not enough to observe the strengthening effect. It is noteworthy that the tensile steel bars were still under elastic state after preloading. The material properties and dimensional details of the test specimens are shown in Table 2 and Figure 5. All specimens, including the control specimen, have rectangular sections of 200 mm in width and 315 mm in height. The total length of specimens was 3500 mm, and their span lengths were 3000 mm. The longitudinal tension and compression reinforcements were 3-D22 and 2-D10, respectively, and shear reinforcement consisted of two legged D10 stirrups at a spacing of 150 mm. The specimens except for the control specimen were damaged in advance through preloading, and they were then strengthened by the section enlargement method by 50 mm in member height. Also, two D13 reinforcements were placed along the longitudinal direction at the enlarged section.

**Table 2:** Characteristics and material properties of specimens *f_ck_*: compressive strength, *f_sp_*: splitting tensile strength, *f_r_*: modulus of rupture, *f_yl_*: tensile strength of longitudinal steel bars, *f_yt_*: tensile strength of transverse steel bars.

Specimen	*f_ck_* (MPa)	*f_sp_* (MPa)	Material	Longitudinal reinforcement						Transverse reinforcement	
				Steel bars	*f_yl_* (MPa)	Steel bars	*f_yl_* (MPa)	Steel bars	*f_yl_* (MPa)	Steel bars	*f_yt_* (MPa)
GB0-00	34.39	2.86	–	3-D22	421	–	–	2-D10	486	D10	486
ABA-65	33.89	3.07	In-place concrete with Anchors	3-D22	505	2-D13	503	2-D10	486	D10	486
GBG-65	33.18	3.60	HCGS hybrid	3-D22	505	2-D13	503	2-D10	486	D10	486
GBI-65	30.75	3.04	HCGS	3-D22	505	2-D13	503	2-D10	486	D10	486

As shown in Figure 5, specimen GB0-00 was the control specimen without strengthening. Specimen ABA-65 has sufficient horizontal shear resistance due to inserting chemical anchors and pouring concrete onto the interface between the base member and the enlarged sectional part, which is a typical method used in practice for application of the section enlargement method. Each anchor was arranged at a spacing of 300 mm, and the chemical anchors used in this study were Hilti-M12 [34], whose physical properties are shown in Table 3. Specimen GBG-65 was strengthened by the section enlargement method applying the HCGS hybrid, *i.e.*, a 1:3 mixture of HCGS and cement paste, onto the new enlarged section, for which no anchor was used. Specimen GBI-65 was strengthened by the section enlargement method using a precast reinforced concrete panel element, in which the prefabricated panel was directly attached to the damaged member by using HCGS binding material, as shown in Figure 5c. Because pouring cast-in-place concrete to the bottom of a beam is a highly tricky job at the construction site, the panel strengthening method was developed to improve the constructability of the section enlargement method and applied to Specimen GBI-65 in this study. The precast panel was made of concrete with the same mixture used for Specimen GB0-00, and was cured for 28 days at the precast factory.

The design compressive strengths of the test specimens were 30.0 MPa. Type-I Portland cement was used, and the maximum coarse aggregate size was 20.0 mm; ϕ100 × 200 mm cylinders were also cast and cured under the same conditions with the beam specimens. The uniaxial tension tests on steel reinforcements showed that the average yield strength was 505, 503, and 486 MPa for D22, D13, and D10, respectively. The material properties of the concrete, HCGS hybrid, and reinforcements measured from material tests are summarized in Table 2. The compressive and splitting tensile strengths (*f_ck_* and *f_sp_*) of the concrete before strengthening were 34.4 MPa and 2.86 MPa, respectively, and those of HCGS hybrid were 33.9 MPa and 3.60 MPa, respectively. It is noteworthy that the tensile strength of the HCGS hybrid is higher than that of conventional concrete with a similar compressive strength.

### 3.2. Loading and Measurements

**Figure 6:**
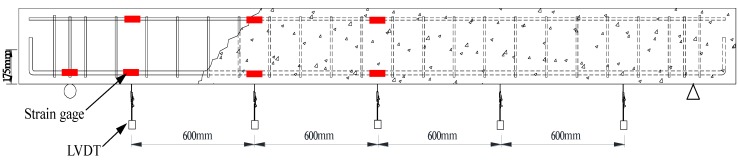
Locations of the gages and linear variable displacement transducers (LVDTs).

As shown in Figure 6, strain gauges were installed to measure the strain behavior of the compression and tension reinforcements, which were located 600 and 1200 mm from the center of the specimens. In addition, to measure the deflection of the test specimens, linear variable displacement transducers (LVDTs) were installed symmetrically around the center of the specimen at the same locations as the strain gauges. Single point loading was applied to the mid-span of test specimens, and the tests were terminated when the load was dropped below 80% of the maximum load. All the measured data from the experiments were collected every second by the data logger.

**Table 3:** Properties of chemical anchor *Ɩ_p_*: length of capsule (mm), *d_p_*: diameter of capsule (mm), *Ɩ*: length of capsule (mm), *A_s_*: stress area (mm^2^), *f_uk_*: tensile strength (N/mm^2^), *f_yk_*: yield strength (N/mm^2^), *W*: resisting moment (mm^3^), *M_Rd,s_*: bending resistant (N·m), *S_w_*: diameter of nut (mm), *d_w_* diameter of washer (mm).

Type	Mechanical/Physical Properties									
Anchor	*Ɩ_p_*	*d_p_*	*Ɩ*	*A_s_*	*f_uk_*	*f_yk_*	*W*	*M_Rd,s_*	*S_w_*	*d_w_*
	110	13.1	160	76.2	500	400	93.9	45.1	19	24

### 3.3. Load-Deflection Behavior of the Test Specimens

**Figure 7:**
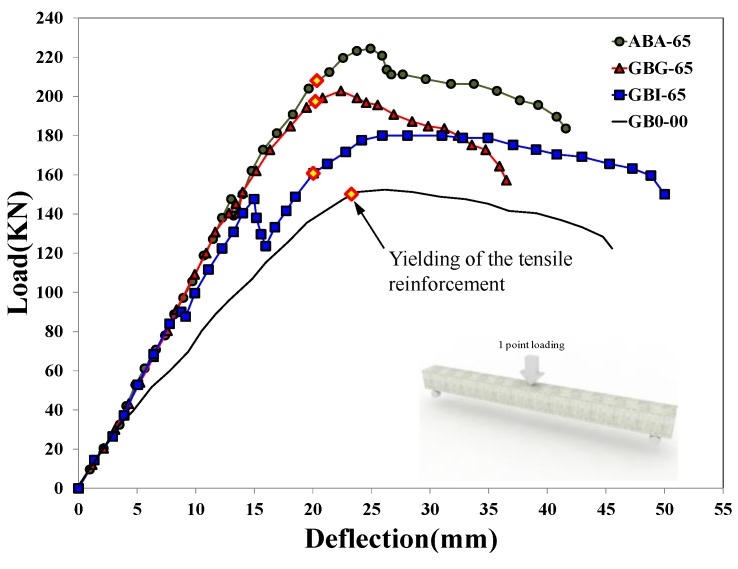
Load-deflection responses of test specimens.

**Figure 8:**
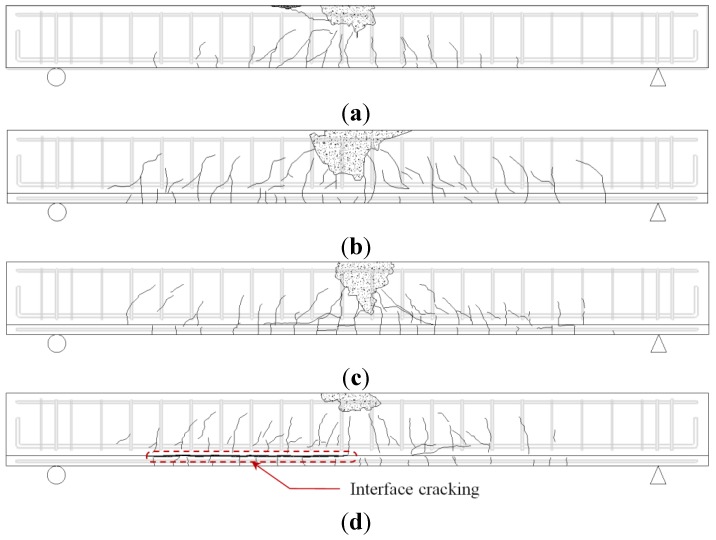
Crack patterns of specimens at failure; (**a**) GB0-00; (**b**) ABA-65; (**c**) GBG-65; (**d**) GBI-65.

The load-deflection responses of the specimens are shown in Figure 7, and their observed crack patterns in Figure 8. The initial flexural stiffness, which is calculated as the slope of the load-deflection curve in the range of 0–70 kN, of the strengthened specimens (ABA-65, GBG-65, and GBI-65) increased approximately up to 1.5 times that of the control specimen (*i.e.*, GB0-00 specimen), and their flexural capacities were also enhanced compared to the control specimen. All specimens showed typical flexural behavior, where the longitudinal tension reinforcement yielded, followed by sufficient ductile behavior and crushing of the concrete at the extreme compressive fiber near the maximum moment region immediately before failure.

Specimen ABA-65, which was typically strengthened by using chemical anchors at the interfaces between the bottom of the damaged concrete member and the cast-in-place concrete, demonstrated the highest flexural strength and stiffness with an increase of the member strength of approximately 47% compared to the control specimen GB0-00. This excellent performance was due to the sufficient horizontal shear capacity provided by the shear connectors installed at the interface, which led to almost fully composite behavior. In the case of GBG-65 specimen, although no anchor was installed, it showed similar behavior to specimen ABA-65 prior to its maximum load, which was possible because of the excellent bonding performance of the HCGS hybrid. Specimen GBG-65 demonstrated a slightly lower flexural strength compared to specimen ABA-65, but its flexural capacity was enhanced by 32% compared to the control specimen. In the case of the GBI-65 specimen strengthened by the panel strengthening method, cracking was observed at the interface between the beam member and the attached panel at a load of 88.2 kN. This was later followed by interfacial separation at the load level of 150.0 kN accompanied by substantial deformation, sharp load reduction, and stiffness degradation. Afterwards, however, the load increased again even after separation at the interface, and the maximum load increased by approximately 18% compared to the control specimen. If the separation at the interface had occurred when the load was close to the maximum strength of the strengthened member, the load recovery could have not been shown. Specimen GBI-65, however, experienced interfacial separation before the yielding of the steel bars, and the specimen was still under elastic range. Thus, both the upper and the lower members still had their remaining flexural capacities, even if they showed non-composite action at the loading stage. The poor application of the HCGS at the interface was due to the unevenness of the bottom surface of the damaged beam, which led to horizontal shear failure at the interface. Although the pre-deflection and small fabrication errors of the damaged member were very similar in all specimens, it was not a problem for other specimens because they were strengthened by pouring cast-in-place concrete or the HCGS hybrid directly onto the bottom surface of the damaged member. For specimen GBI-65, however, the concrete panel was fabricated separately, whose surface was very flat, and it was attached onto the damaged member with pre-deflection by a thin layer of HCGS. Of course, as this can happen in practice, it is an important issue for the panel strengthening method to function. Therefore, further research and complementary measures should be addressed for the application of the panel strengthening method. On the contrary, in the case of GBG-65 specimen strengthened by cast-in-place HCGS hybrid, no interfacial separation was observed even at the maximum load, showing only a few fine interfacial cracks which occurred immediately before completion of the experiment. Specimen ABA-65 strengthened with chemical anchors had no interfacial separation. Thus, it can be mentioned with care that the application of the panel strengthening method with a small number of anchor bolts could be an adoptable strengthening method to improve constructability.

## 4. Evaluation of the Test Results

### 4.1. Partial Interaction Theory (PIT)

To quantitatively evaluate the flexural performances and the degree of connections of the specimens strengthened by the section enlargement method, in this study, flexural analyses were conducted based on partial interaction theory (PIT) [35,36,37]. According to the PIT presented by Newmark [38], the degree of connections of steel-concrete composite members can be determined by the horizontal shear capacities and shear stiffness of the interfacial shear connection [39,40]. Due to the inter-related complex mechanisms, the basic assumptions described below were introduced to simplify the analysis of the concrete composite members strengthened by the section enlargement methods [35,41]:

(1) Concrete is a homogeneous and isotropic material;
(2) The shear connection at the interface (bonding at interface) between composite components is continuous and constant along the longitudinal direction of the member;
(3) The base and the expanded sections remain in plane even after flexural deformation, and their curvatures are the same;
(4) Separation in a vertical direction does not occur, and thus only shear stress is taken into account for the analysis.

**Figure 9:**
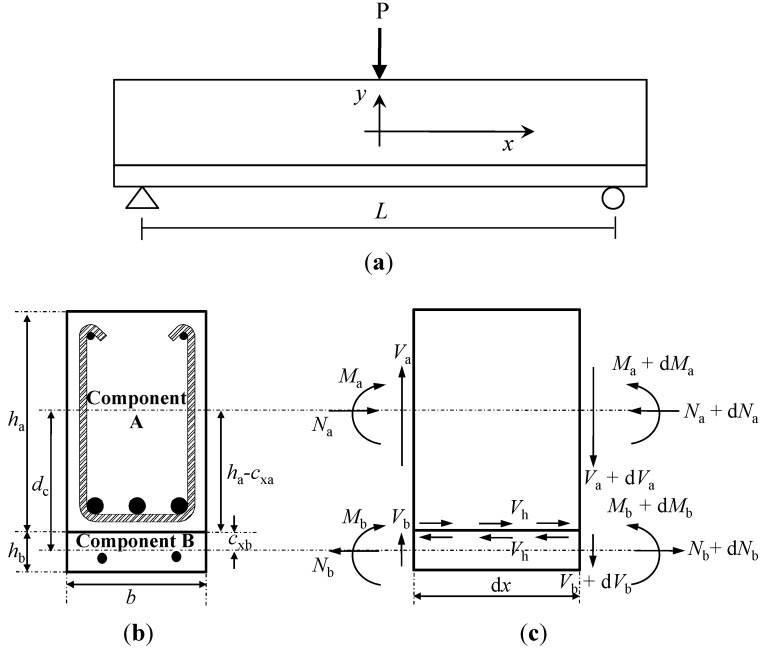
Force equilibrium in a section of composite beam strengthened by section enlargement; (**a**) simply-supported composite beam; (**b**) cross section; (**c**) force elements.

Based on these assumptions, as shown in Figure 9, the force equilibrium equations in the horizontal and vertical direction can be derived, respectively, as follows:

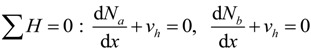
(1)

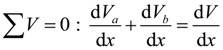
(2)
where, Σ*H* and Σ*V* are the sum of the horizontal and vertical forces, respectively, *N_a_* and *N_b_* are the axial forces acting on the sections of the base member (component A) and the enlarged part (component B), respectively, *ν_h_* the horizontal shear stress at the interface, *V_a_* and *V_b_* are the shear forces on the sections of the base member and the enlarged part, respectively, and *V* is the shear force acting on the section of the composite member. The moment equilibrium equation for the components A and component B can be expressed, respectively, as follows:

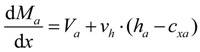
(3a)

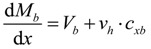
(3b)
where, *M_a_* and *M_a_* are the flexural moments developed in the components A and B, respectively, *h_a_* is the sectional height of the component A, *c_xa_* and *c_xb_* are the depths of the neutral axis in the components A and B, respectively. By summing Equations 3a and 3b and differentiating both sides with respect to the variable *x*, the differential equation can be derived, as follows:

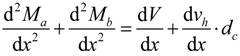
(4)
where, *V* = *V_a_* + *V_b_* and *d_c_* = (*h_a_* − *c_xa_* + *c_xb_*). In addition, the total horizontal shear force (*V_h_*) at the interface of the composite member acting across the shear span (*a*) can be estimated, as follows:

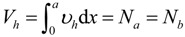
(5)


The horizontal shear force is equal to the axial force in the components A or B (*i.e.*, *V_h_* = *N_a_* = *N_b_*). Therefore, by integrating both sides of Equation 4, the flexural moment of the composite section strengthened by the section enlargement method can be obtained, as follows:

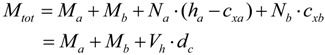
(6)
where, *M_tot_* is the flexural moment acting on the composite section. In other words, the flexural strength of the composite member considering the strengthening effect can be expressed as a function of the interfacial horizontal shear force and the flexural strengths of the components A and B. Thus, it is clear that the horizontal shear capacity at the interface determines the degree of strengthening efficiency provided by the section enlargement method.

### 4.2. Analysis Methodology

**Figure 10:**
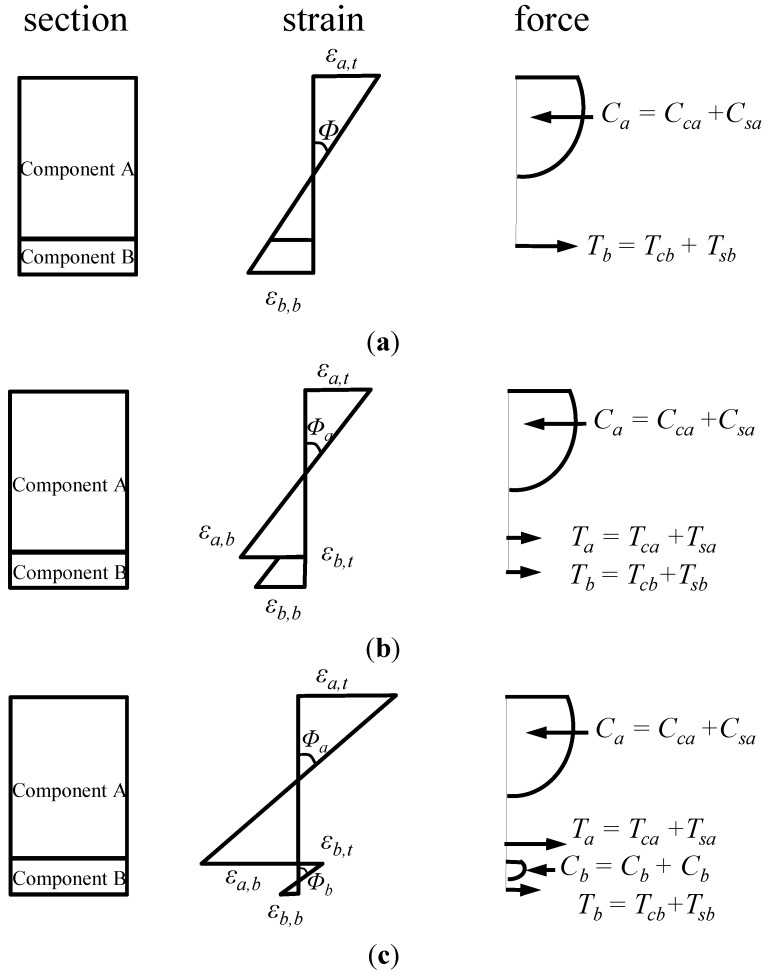
Strain and force distributions of composite beams according to interface conditions; (**a**) perfect connection; (**b**) partial connection; (**c**) no connection.

**Figure 11:**
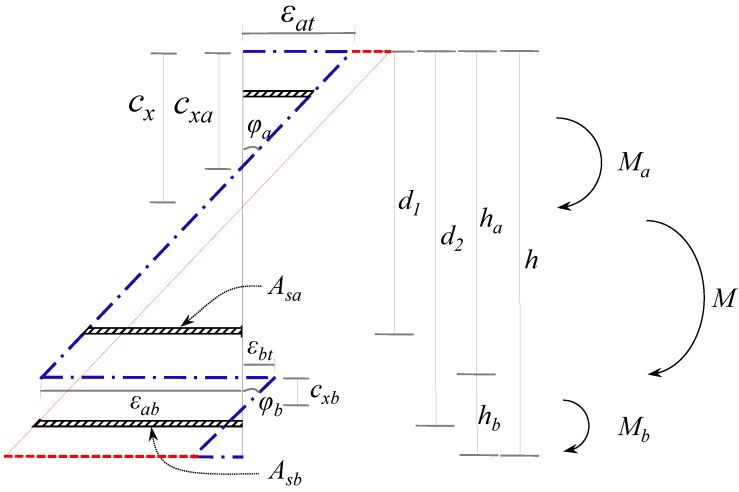
Sectional strain distribution in the analytical approach.

**Figure 12:**
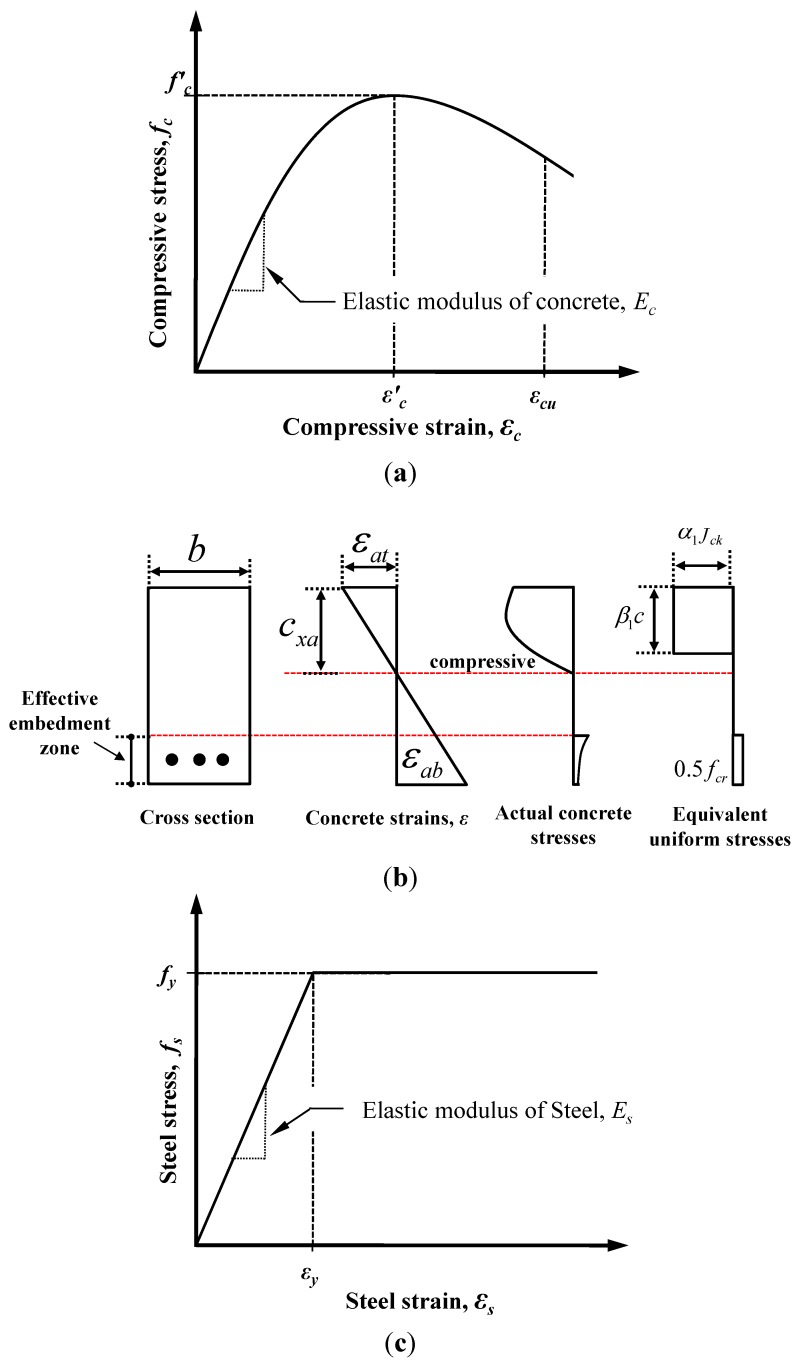
Material models for concrete and reinforcement adopted in the partial interaction theory (PIT)-based analysis; (**a**) concrete; (**b**) stress-block factors; (**c**) reinforcement.

Figure 10 shows the distributions of the strains and forces in the section of the composite member strengthened by the section enlargement method according to the conditions of interface connections. Figure 10a–c show a full interaction, a partial interaction, and a non-composite action, respectively. As expressed in Equation (6), the flexural behavior of the composite member can be evaluated by adding the moment contribution due to the horizontal shear force at the interface to the sum of the flexural moments developed in the components A and B. In other words, the flexural moments of the components A and B that experienced the same curvature were calculated by assuming a non-composite behavior, as indicated by the blue one-dotted chain line in Figure 11, on which the flexural moment contribution provided by the horizontal shear force required for a complete composite action, as depicted by the red dotted line in Figure 11, was added to estimate the total flexural resistance of the composite section. Note that the horizontal shear strength can be determined by Equation (15). The sectional analysis of the composite members strengthened by the section enlargement method starts from selecting the extreme top and bottom strains of the component A (*ε_at_* and *ε_ab_*). Once *ε_at_* and *ε_ab_* of the component A are determined, the neutral axis depth (*c_xa_*) and the strains in the steel reinforcement of the component A can be determined. Then, the compression force in concrete (*C_ca_*), the compressive force in the compression reinforcement (*C_sa_*), the tension force in concrete (*T_ca_*), and the tension force in the steel reinforcement (*T_sa_*) can be easily calculated for the component A. As for the concrete stress-strain relationship, the model of Collins *et al.* [42] shown in Figure 12a was used, and the compression force of concrete was calculated by the equivalent stress factor [42], as shown in Figure 12b. A more detailed calculation process based on the equivalent stress factor model can be also found in other references [43,44,45]. The stress-strain relationship of the steel reinforcement was assumed to be bi-linear behavior [46], as shown in Figure 12c. The force equilibrium condition of component A in Figure 10 can be expressed, as follows:

Σ*F_xa_* = *C_sa_* + *C_ca_* + *T_sa_* + *T_ca_* (7)

where, Σ*F_xa_* is the resultant force of the component A, and *C_sa_* and *T_sa_* are the compressive and tension forces in the steel reinforcements, respectively. In addition, *T_ca_* can be determined according to the strain level of extreme bottom fiber of the component A (*ε_ab_*), as follows [42]:

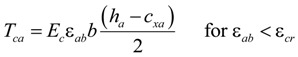
(8a)

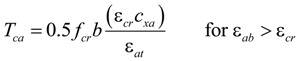
(8b)
where, ε*_cr_* and *f_cr_* are the cracking strain and strength of the concrete. If the force equilibrium condition is satisfied in the section considered, the flexural resistance of the component A (*M_a_*) can be calculated, as follows:

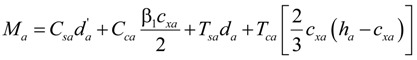
(9)
where, *d’_a_* and *d_a_* are the distances from the extreme compressive fiber to the centroid of the compression reinforcement and the tension reinforcement of the component A, respectively. The curvature corresponding to the estimated flexural moment can be calculated, as follows:

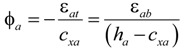
(10)


According to the aforementioned assumption (3), the curvature of the component A is identical to that of the section enlarged part (component B), which can be expressed, as follows:

ϕ*_b_* = ϕ*_a_* (11)

Then, the strain distribution of component B can be determined by assuming the depth of the neutral axis (*c_xb_*). Based on the same procedure as carried out for component A, the equilibrium condition of the sectional forces in component B can be obtained, as follows:

Σ*F_xb_* = *C_sb_* + *C_cb_* + *T_sb_* + *T_cb_* (12)

If this equilibrium condition is not satisfied, the sectional forces and strains of component B should be calculated again by changing the assumed neutral axis (*c_xb_*). Here, *C_cb_* is the compression force of the concrete, *C_sb_* is the compressive force by the compression reinforcement, *T_cb_* is the tension force provided by the concrete, and *T_sb_* is the tension force in the tension reinforcement of component B. If the force equilibrium is satisfied, the flexural moment of component B (*M_b_*) can be estimated, as follows:

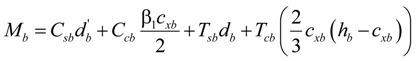
(13)
where, *d’_b_* and *d_b_* are the distances from the extreme compression fiber to the centroid of the compression reinforcement and tension reinforcement of component B.

In order to determine the flexural moment of the composite members strengthened by the section enlargement method, the horizontal shear force (*V_h_*) should also be estimated in addition to *M_a_* and *M_b_*, as shown in Equation (6). The horizontal shear force (*V_h,req._*) required to achieve the full-interaction conditions at the interface between components A and B is equal to the tension force in component B. The tensile contribution of the concrete can be ignored because it is relatively very small, and the required horizontal shear force (*V_h,req._*) can be calculated, then, as follows:

*V_h,req._* = *T_cb_* + *T_sb_* ≈ *T_sb_* = ϕ*_a_* (*d_2_* − *c_x_*)*E_s_A_sb_* (14)

where, *c_x_* is the depth of the neutral axis of the composite section, and *A_sb_* is the sectional area of the tension reinforcement placed in component B. As aforementioned, in case the horizontal shear strength at the interface (*V_h,n_*) was greater than the required horizontal shear force (*V_h,req._*), the horizontal shear force acting at the interface (*V_h_*) was assumed to be identical with the required horizontal shear force (*V_h,req._*). On the other hand, in case the horizontal shear force required at interface (*V_h,req._*) was greater than the horizontal shear strength (*V_h,n_*), the horizontal shear force (*V_h_*) at the interface was assumed to maintain the horizontal shear strength (*V_h,n_*) only. Thus, the horizontal shear force (*V_h_*) acting at the interface can be expressed, according to the level of shear force, as follows:

*V_h_* = *V_h,req._*  when  *V_h,n_* ≥ *V_h,req._* (15a)

*V_h_* = *V_h,n_*   when  *V_h,n_* ˂ *V_h,req._* (15b)

**Figure 13:**
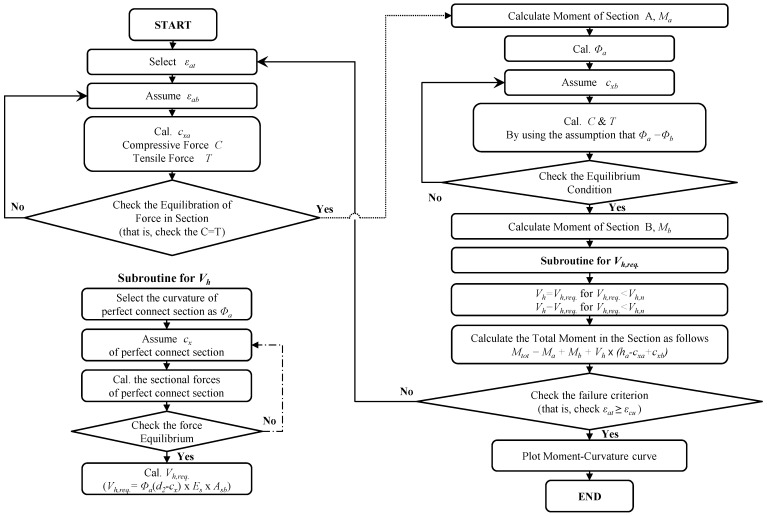
Flowchart for the PIT-based analysis.

When the horizontal shear force (*V_h_*) is determined, it is substituted in Equation (6) to get the flexural moment of the composite section. The flexural moment-curvature responses of the composite members strengthened by the section enlargement method can be determined by repeating the aforementioned calculation processes by increasing the top and bottom fiber strains of the composite section until the top fiber strain reaches the ultimate compressive strain of the concrete (ε*_cu_*). Figure 13 shows the calculation procedures on flexural behavior analysis introduced in this study.

### 4.3. Comparison of the Analysis and Experimental Results

**Figure 14:**
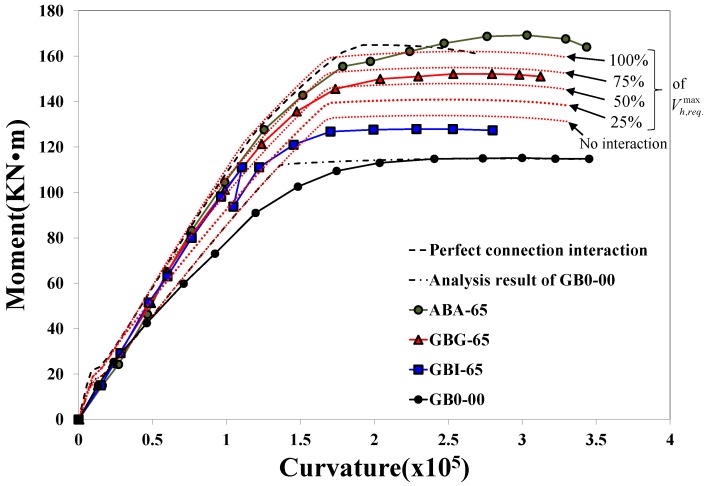
Comparison of flexural moment-curvature responses estimated by PIT-based analysis and test results.

The degrees of connection and flexural behavior of specimens were quantitatively evaluated using flexural behavior analysis described in the previous section. In this study, comprehensive analyses were performed for the cases where the horizontal shear strength (*V_h,n_*) was 100, 75, 50, and 25% of the maximum horizontal shear force (*V_h,req._*), respectively. The maximum horizontal shear force required (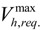) can be estimated, as follows:

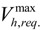
 = *A_sb_f_sb,y_*(16)
where, *f_sb,y_* is the yield strength of the tension reinforcement placed in component B. Figure 14 compares the flexural behaviors of specimens observed from the experiments and those calculated by the PIT-based analysis. Note that the curvatures of test results were calculated from the strains measured by gages, and the curvature after the peak could not be presented in Figure 14 due to the limited range of the gage measurement, while the post-peak load-deflection responses are shown in Figure 7. The red dotted lines in the graph represent the analysis results of specimens according to the different levels of interactions, and the black dotted line represents the analysis results estimated by assuming full interaction (refer to Figure 10a). The graph expressed by a one-dotted chain line represents the results of flexural behavior analysis on the non-strengthened specimen GB0-00. It is apparent that the moment-curvature behavior analyzed with the assumption that the maximum horizontal shear force (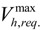) can be entirely transmitted at the interface was almost consistent with that obtained by assuming full composite behavior. In addition, the analysis result obtained by assuming non-composite behavior (*i.e.*, 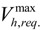 = 0) showed approximately 20% higher strength and similar initial flexural stiffness compared to the non-strengthened specimen GB0-00. It should be noted that specimen GB0-00 had steel bars with a lower yield strength as indicated in Table 2, and, if their yield strength had been the same as the steel bars used in other specimens, the moment capacity would have been even closer to that of the non-composite section.

The initial flexural stiffness of all the strengthened specimens showed similar behavioral characteristics as shown in Figure 7 as well as Figure 14. The flexural response of the non-strengthened specimen GB0-00 estimated by the PIT-based analysis showed good agreement with the test result. It is also shown that the observed maximum moment capacity of specimen GBG-65 is very similar to that estimated by the analysis with the condition of 75% interaction ratio, *i.e.*, 0.75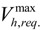. In addition, the flexural moment of specimen GBI-65 strengthened by the panel strengthening method decreased temporarily due to severe interfacial cracking occurring at 112.5 kN·m, and then it showed similar behavior to the analysis results estimated by the analysis assuming 25% shear capacity of 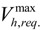. Afterward, its behavior became similar to the analysis result with non-composite action at the interface (*i.e.*, 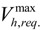 = 0). In this case, *i.e.*, the non-composite behavior, about 15.0% of the flexural capacity was enhanced by structural strengthening compared to the control specimen.

Among the test specimens strengthened by the section enlargement method, specimen ABA-65 strengthened by concrete with shear anchors showed the closest behavior to the analysis results based on full interaction behavior. It is also shown that specimen GBG-65, strengthened by the HCGS hybrid without any shear connector, was evaluated as capable of transmitting about 70% horizontal shear of the maximum shear force (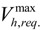) required at the interface. This result indicates that the amount of shear connectors (or anchors) can be significantly reduced for structural strengthening using the HCGS hybrid (specimen GBG-65), and it is expected that the constructability of strengthening work can be greatly improved. Specimen GBI-65 strengthened by the panel strengthening method showed improved initial stiffness compared to the non-strengthened specimen GB0-00, but poorer flexural behavior at the ultimate state, similar to the non-composite member analysis result, in which the complete separation behavior was assumed, *i.e.*, 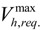 = 0. Accordingly, in order to use the panel strengthening method in practice, additional considerations would yet be necessary for the proper application of the HCGS to the interface of the damaged member depending on the flatness condition of the bottom surface. Although there were some differences between the observed values of the ultimate curvatures and the analysis results, it is to be noted that the PIT-based analysis is meaningful because it gives an indication of the levels of compositeness of the strengthened members.

## 5. Conclusions

In this study, reinforced concrete beam members were initially damaged, and then strengthened by the section enlargement method utilizing HCGS developed to overcome the limitations of existing binding materials for structural strengthening. In addition, experiments on four test specimens were conducted, and their flexural responses were analyzed by PIT-based analysis considering the degree of connection between the base section and the enlarged part. On this basis, the following conclusions can be drawn:

(1) The HCGS introduced in this study showed superior basic material characteristics compared to existing epoxy binding materials. In particular, a more powerful binding structure was established by forming a Si-O ionic bond between the cement hydration products within the concrete, leading to excellent bond performance and durability.
(2) Specimen ABA-65, strengthened by the section enlargement method using shear connectors showed the most enhanced flexural capacity, and specimen GBG-65 strengthened by the section enlargement method utilizing the HCGS hybrid showed somewhat lower maximum load but similar flexural behavior with specimen ABA-65 due to the superior bond performance of the HCGS even though no shear connector was provided at the interface.
(3) In specimen GBI-65, strengthened by the panel strengthening method, interfacial separation (*i.e.*, horizontal shear failure) was observed, and only minor strengthening effect was obtained, compared to the other strengthened specimens. It is expected, however, that constructability can be enhanced by proper surface treatment or placing a small amount of shear connectors.
(4) Flexural behavior analyses on the test specimens were performed using PIT-based flexural behavior analysis. This analysis method reasonably reflected the effect of horizontal shear developed at the interface. On this basis, the degrees of connections of the composite members were able to be estimated quantitatively.
(5) In the analysis of the specimens, it was found that specimen ABA-65 showed behavior close to full interaction, and specimen GBG-65 showed about 70% of the horizontal shear capacity of the fully composite member.
